# Thrombolysis for massive pulmonary embolism in pregnancy: a case report

**DOI:** 10.1186/1865-1380-4-69

**Published:** 2011-10-31

**Authors:** Sergio Fasullo, Giorgio Maringhini, Gabriella Terrazzino, Filippo Ganci, Salvatore Paterna, Pietro Di Pasquale

**Affiliations:** 1 Division of Cardiology, "Paolo Borsellino" G.F. Ingrassia Hospital, Palermo, Italy; 2 Department of Emergency Medicine, University of Palermo, Palermo, Italy

## Abstract

Mortality from pulmonary embolism (PE) in pregnancy might be related to challenges in targeting the right population for prevention. Such targeting could help ensure that the correct diagnosis is suspected and adequately investigated, and allow the initiation of the timely and best possible treatment of this disease. In the literature to date only 18 case reports of thrombolysis in pregnant women with PE have been reported, and showed beneficial effects for both mother and fetus in terms of mortality and complications with acceptable bleeding risks. We present here the case of a pregnant patient with massive PE who underwent successful thrombolysis. A 26-year-old pregnant (at 24 weeks) woman was admitted 4 h after onset of sudden acute dyspnea and chest pain. An immediate electrocardiogram showed a typical S1-Q3-T3 pattern. The echocardiogram showed a distended right ventricle with free-wall hypokinesia and displacement of the interventricular septum toward the left ventricle. Thrombolysis with recombinant tissue plasminogen activator (alteplase 10 mg bolus, then 90 mg over 2 h) was administered. Pelvic examination and ultrasound showed regular fetal heart beat, and regular placental and liquid presence. No problems developed for the mother or fetus in the subsequent days or at discharge. In conclusion, in pregnant patients with life-threatening massive PE, thrombolytic therapy can be administered, and the use of echocardiographic, laboratory, and clinical data can be useful tools to achieve a rapid diagnosis and make a therapeutic decision, but additional studies need to be performed to further define its use.

## Introduction

Massive pulmonary embolism (MPE) is the leading cause of maternal mortality in the developed world. Mortality from PE in pregnancy might be related to challenges in targeting the right population for prevention. Such targeting could help ensure that the correct diagnosis is suspected and adequately investigated, and allow the initiation of the timely and best possible treatment of this disease. Thrombolytic drugs can be considered for the treatment of patients who are hemodynamically unstable, or of patients with refractory hypoxemia [1] or right ventricular dysfunction on echocardiogram [2,3]. However, the high risk of major bleeding (in 4%-14% of treated patients with thrombolysis) limits their use [4]. Although pregnancy-specific complications do arise, including spontaneous pregnancy loss, placental abruption, and preterm labor, it is not clear whether they are caused by the underlying disease, its treatment, or neither. We present here the case of a pregnant patient with massive PE (MPE) who was hospitalized 4 h after onset of sudden acute dyspnea and chest pain, and successfully thrombolysed.

## Case report

A 26-year-old pregnant (at 24 weeks) woman was referred to the emergency department (ED) of our hospital ("G.F. Ingrassia" Palermo, Italy) 4 h after onset of sudden acute dyspnea and chest pain. No risk factors or drug consumption was present in the patient's clinical history. On admission to the ED, the patient was dyspneic, cyanotic, hemodynamically unstable, hypotensive (70/50 mmHg), and tachycardic (125 beats/min), with low oxygen saturation (80%) in oxygen with a Venturi mask (6 L/min), with a respiratory rate of 28-30 breaths/min, and with primary hypoxemia and metabolic acidosis (pH 7.29; PO_2 _51 mmHg, PCO_2 _30 mmHg, HCO_3 _20 mmol/L).

**Figure 1:**
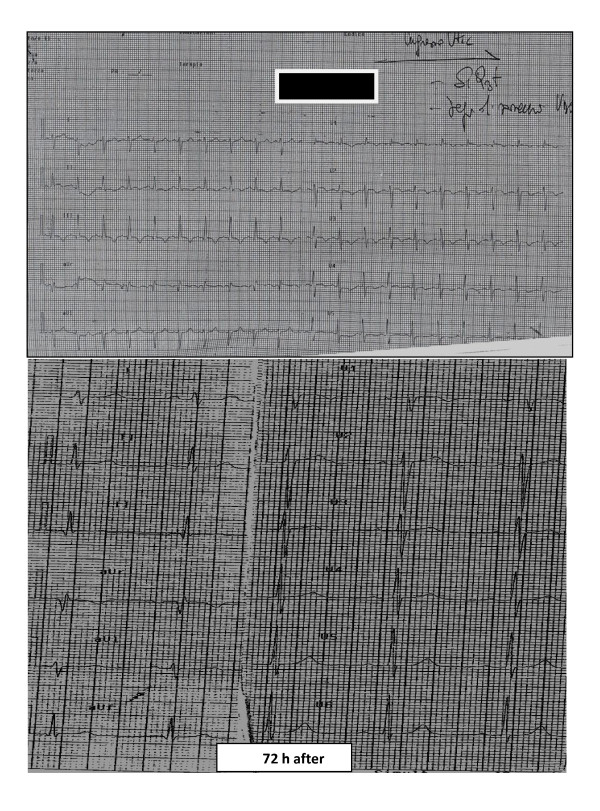
**ECG on admission, before thrombolysis and 72 h after thrombolysis**.

**Figure 2:**
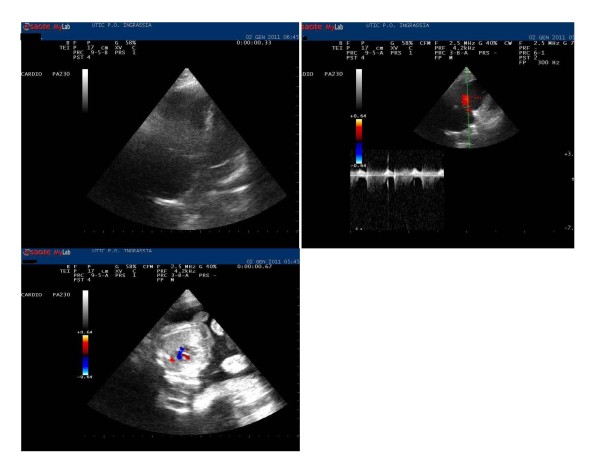
**On admission: right ventricular dysfunction and fetus echocardiogram 4 h after thrombolysis**.

Immediate electrocardiogram showed sinus tachycardia with a typical S1-Q3-T3 pattern (Figure 1). After first aid consisting of intravenous line placement, oxygen treatment, and fluid infusion, the patient was transferred to the cardiology department with a diagnosis of MPE complicated by shock. Plasma samples were obtained to check laboratory parameters including troponin I, prothrombin time, activated partial thromboplastin time, INR, fibrin degradation products, D-dimers, and fibrinogen and N-terminal pro brain natriuretic peptide plasma levels, which were controlled every 6 h for the first 24 h, then every 12 h until clinical stabilization and every 24 h subsequently (Table 1). The echocardiogram performed on admission showed a normally contracting left ventricle, a distended right ventricle with free-wall hypokinesia, and displacement of the interventricular septum toward the left ventricle. In addition, a severe tricuspid regurgitation, pulmonary arterial hypertension (acceleration time < 90 ms with bifid pattern), and inferior vena cava dilatation (26 mm) were present (Figure 2). Spiral computed tomography was not performed because of the pregnancy, and for the same reason, catheter embolectomy was not used. To decide on the diagnosis and treatment, we used only clinical, laboratory, and echocardiographic findings. In a patient with suspected MPE who is in critical condition, bedside echocardiography is particularly helpful in emergency management decisions [3], but in the present case, the presence of pregnancy discouraged performing invasive imaging tests or treatments. Although there was a relative contraindication to thrombolysis [5], it was no longer relevant in the face of an extremely life-threatening situation for the mother and fetus. After informing the patient and obtaining written consent, thrombolytic treatment was carried out with rtPA (10 mg bolus, then 90 mg over 2 h) and a heparin bolus (5, 000 IU) with subsequent heparin infusion (1, 000 U/h), or according to partial thromboplastin time for the first 48 h, when LMWH (enoxaparin 6, 000 IU twice daily) was started [6]. Arterial blood gas evaluation was also performed every 30 min after thrombolytic treatment, and then every 6 h up to stabilization. An improvement in oxygen saturation (> 90%), an increase in blood pressure, a reduction in heart rate, a complete absence of cyanosis, and a reduction in dyspnea 30 min after thrombolysis were observed. Two hours after thrombolysis, we observed a heart rate < 100 beats/min, 98% saturation, pH 7.39; PO_2 _95 mmHg, PCO_2 _34 mmHg, HCO_3 _23 mmol/L, and a blood pressure of 95/60 mmHg. The same day (4 h after thrombolysis), a pelvic examination was performed, and ultrasound showed a regular fetal heart beat, regular placental and normal liquid presence (Figure 1). A gynecological visit and ultrasound control were carried out two times/day (morning and afternoon) until discharge. In addition, we observed an increase in TNI (3.7 pg/ml) and BNP (375 pg/ml), which returned to the normal range 72 h after thrombolysis. The subsequent day, ultrasonography did not show any vein thrombosis. Echocardiogram repeated again 24 to 48 h from thrombolysis showed a clear improvement of the hemodynamics of the right ventricle, disappearance of dilatation, normalization of pulmonary pressures, normalization of septal motion, and reduction of vena cava diameter (20 mm after 48 h and 16 mm after 72 h). The S1-Q3-T3 was no longer present in the electrocardiogram 72 h after thrombolysis (Figure 1). On the 5th day, the patient was transferred from intensive care and discharged 8 days after. No problems developed in the subsequent days for the mother and fetus, which was controlled every day and before discharge. In the first 36 h we observed a modest Hb reduction (about 1 g), and the plasma level of fibrinogen in plasma was very low, almost undetectable.

**Table 1:** Clinical and laboratory parameters in the first 72 h after admission.

	Entry	2 h	72 h
BP mmHg	70/50	95/60	110/70
HR beats/min	125	98	82
OS (6 L/min O2)	80%	98 (6 L/min O_2_)	99% room air
RR breaths/min	28-30	22	16
pH	7.29	7.39	7.44
PO_2 _mmHg	51	95	99
PCO_2 _mmHg	30	34	40
HCO_3 _mmol/L	20	23	24
ECG	S1-Q3-T3		Disappeared
TNI pg/mL	3.7		< 0.02
BNP pg/mL	375		< 100

**Figure 3:**
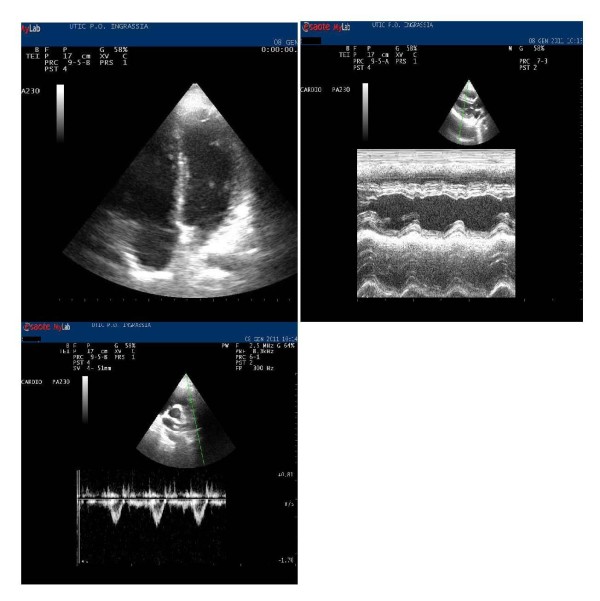
**Predischarge (7 days): right ventricle function normalization**.

BP, blood pressure; HR, heart rate; OS, oxygen saturation; RR, respiratory rate.

and the plasma fibrinogen was undosable, while the other hematological parameters were in normal range. No blood transfusions were required. The plasma fibrinogen returned to the normal range 48 h after thrombolysis. The hemoglobin increased in the subsequent days up to 11.5 g. No minor or major bleeding was observed, and the placental and fetal examination was always normal. All laboratory parameters were normalized at discharge. In addition, during hospitalization a selective study of the coagulation at the hematological clinic of the University of Palermo was also performed, and important alterations were not found. The patient was discharged and underwent LMWH treatment (Figure 3). The patient is being followed up at our outcome clinic, together with a gynecologist, to evaluate the fetal status and develop subsequent strategies, also for postpartum.

## Discussion

Women who are pregnant or in the postpartum period as well as women receiving hormonal therapy are at increased risk for venous thromboembolism. Venous thromboembolism is responsible for up to 15% of all in-hospital deaths, and it also accounts for 20% to 30% of deaths associated with pregnancy and delivery in the United States and Europe. In pregnant patients with suspected acute PE, the use of noninvasive diagnostic methods without imaging may seem ideal, but concern about exposure to radiation should not deter clinicians from using computed tomography angiography or ventilation-perfusion scanning when necessary. Although experience with thrombolytic therapy in pregnancy is limited (only 18 cases treated with different thrombolytic drugs have been reported), the use of thrombolytic agents may be lifesaving in patients with MPE and severe hemodynamic compromise [7-14]. In these 18 case reports of pregnant women treated with systemic thrombolysis for MPE, the most commonly used regimen during pregnancy was 100 mg tPA over 2 h (10 patients), while 6 patients received STK and 2 urokinase. Concerning complication rates in pregnant women (major nonfatal bleeding), only 4 of 18 cases were observed in the streptokinase group. In addition, preterm delivery occurred in two patients with tPA and three in the streptokinase group. Two child deaths were reported (1 in the streptokinase and 1 in the tPA group), but they were not attributed to fetal hemorrhage [7-14]. There is concern that thrombolytic therapy will lead to placental abruption, but this complication has not been reported. The care of the pregnant patient who has MPE either at term or when suspicion of compromised fetal status calls for expeditious cesarean delivery is complex and requires a coordinated treatment strategy by the obstetrician, intensivist, cardiothoracic surgeon, anesthesiologist, and interventional radiologist. The approach to the management of an MPE should be individualized and adapted to changing circumstances. Although thrombolytic therapy is considered to be (relatively) contraindicated, successful outcomes with the use of thrombolytic therapy during labor have been reported [15,16]. We report the case of a 26-year-old pregnant (at 24 weeks) woman with MPE who was successfully treated with thrombolysis. We used rTPA because this fibrinolytic agent does not cross the placental barrier. We recognize that thrombolysis can be dangerous in the early phases of pregnancy, but the urgency of the case required a quick decision. In addition, we also showed that the echocardiogram and clinical and laboratory parameters were invaluable tools to reach a rapid corrected diagnosis, allowing us also to follow the effects of treatment. This choice avoided using possibly dangerous radiant imaging tools on the fetus. In addition, according to ESC guidelines, in mothers the overall incidence of bleeding is about 8%, usually from the genital tract. This risk does not seem unreasonable compared with the death rate seen in patients with massive PE treated with heparin alone [5]. In conclusion, in a patient with life-threatening PE, thrombolytic therapy should not be withheld solely because of pregnancy, but additional studies need to be performed to further define its use.

## Consent

the consent of the publication of scientific work has been signed.

## Competing interests

The authors declare that they have no competing interests.

## Authors' contributions

SF and PDP conceived of the study. PDP and SP drafted the manuscript. GM, GT and FG participated in the sequence alignment. All authors read and approved the final manuscript.
